# Vertical datum transformation grids for Mexico

**DOI:** 10.1038/s41597-020-0511-x

**Published:** 2020-06-03

**Authors:** Jaime J. Carrera-Hernández

**Affiliations:** Centro de Geociencias, UNAM, Queretaro, Mexico

**Keywords:** Solid Earth sciences, Geography

## Abstract

Mexico has used two vertical datums—the U.S. National Geodetic Vertical Datum of 1929 (NGVD29) and the North American Vertical Datum of 1988 (NAVD88). Because Mexico started using the NAVD88 as its vertical datum in 2015, most of Mexico’s data is referenced to the NGVD29 and recent high resolution data are referenced to the NAVD88. Compounding this situation, satellite-derived Digital Elevation Models (DEMs) are referenced to the Earth Gravimetric Model 96 (EGM96), and no tools are currently available in Mexico to transform elevation data between the aforementioned vertical datums. To overcome this problem, this work presents the development of two surfaces to transform orthometric heights between the NGVD29 and NAVD88 or between the NGVD29 and EGM96 in Mexico. These surfaces can be used by any interested user to transform orthometric heights referenced to the aforementioned datums.

## Abstract (summary)

**Table:** 

Measurement(s)	height above vertical datum
Technology Type(s)	data transformation
Sample Characteristic - Environment	planetary surface
Sample Characteristic - Location	Mexico

Machine-accessible metadata file describing the reported data: 10.6084/m9.figshare.12302564

## Background & Summary

Height is defined as the distance—measured along a perpendicular—between a point and a reference datum. According to the vertical datum of reference used, a given height can be either ellipsoidal, geodetic, or orthometric^1^. Users of Digital Elevation Models (DEMs) should be aware of which datum their data are referenced to because differences between vertical datums can exceed tens of meters^2^. This awareness is important due to the existing difference between local vertical datums and the satellite derived DSMs that provide global coverage and which use the Earth Gravitational Model 1996 (EGM96^3^) geoid as reference surface, such as the Shuttle Radar Topography Mission DSM (SRTM^4^), the Advanced Spaceborne Thermal Emission and Reflectance Radiometer DSM (ASTER^5^) or the more recent Advanced Land Observing Satellite World 3D-30m DSM (AW3D30^6^).

Orthometric heights (*H*) are referenced to a vertical datum of zero elevation, which has normally been considered to be the Mean Sea Level (MSL) adopted from a local tide gage—which explains why there are more than 200 vertical datums used in the world today^7^. MSL was long considered as a reference surface;^1^ however, MSL is not an equipotential surface and other forces besides gravity—temperature, salinity, currents and wind—affect it^7^. This situation means that the zero height determined at one sea-station will not be equal to the zero height determined at a different sea-station. This problem was addressed in North America through the development of two vertical datums: the U.S. National Geodetic Vertical Datum of 1929 (NGVD29) and the North American Datum of 1988 (NAVD88), which are the vertical datums that have been used in Mexico.

Because the concepts involved on defining the ellipsoidal, geodetic, and orthometric heights are given in different publications^1,2,8–11^ only a brief summary is provided in order to show the motivation of this work. The ellipsoidal height (*h*) of a given point represents its distance from a reference ellipsoid measured along a line normal to it—which is the height provided by a Global Navigation Satellite System (GNSS) or Global Positioning System (GPS)—while the geoid height (*N*) represents the difference between an ellipsoidal height (*h*) and an orthometric height (*H*). These three heights are related to each other according to:

1 $$H\approx h-N$$

which shows that the conversion from ellipsoidal to orthometric heights can be done through the use of a geoid height model. Orthometric heights with an accuracy similar to that of leveling surveys can be obtained through the use of careful GNSS/GPS survey procedures coupled with high-resolution geoid models^12^. This is why a new North America Vertical Datum (NAVDXX) has been proposed for 2022, which will be based on GNSS/GPS positioning and a high accuracy geoid that will cover Mexico, Canada and the Conterminous United States^13^. Canada has already updated its vertical datum to the Canadian Geodetic Vertical Datum of 2013 (CGVD2013), replacing the geodetic levelling technique by a geoid model^14^.

Mexico has used both the NGVD29 and the NAVD88 as vertical datums and all maps and databases developed by Mexico’s National Institute of Geography and Statistics (INEGI) before 2015—when the NAVD88 was adopted as Mexico’s current vertical datum^15^—were referenced to the NGVD29. In fact, the elevations of Mexico’s Digital Terrain Model (CEM) created by INEGI are still referenced to the NGVD29, while the new high-resolution topography datasets developed by INEGI through either LiDAR or photogrammetry are referenced to the NAVD88. Compounding this variability, the satellite-derived Digital Surface Models SRTM, ASTER and AW3D30 use the EGM96 as vertical datum. For the Conterminous United States (CONUS), the U.S. National Geodetic Survey developed the VERTCON software^16^ to convert heights between NGVD29 and NAVD88, which have been found to range from −40 to 150 cm^17^. In Mexico there are no tools to convert orthometric heights referenced to either the NGVD29 (*H*_NGVD29_), NAVD88 (*H*_NAVD88_) or EGM96 (*H*_EGM96_). To overcome this issue, this work details the generation of surfaces that can be used to transform orthometric heights between the aforementioned vertical datums.

## Methods

**Fig. 1:**
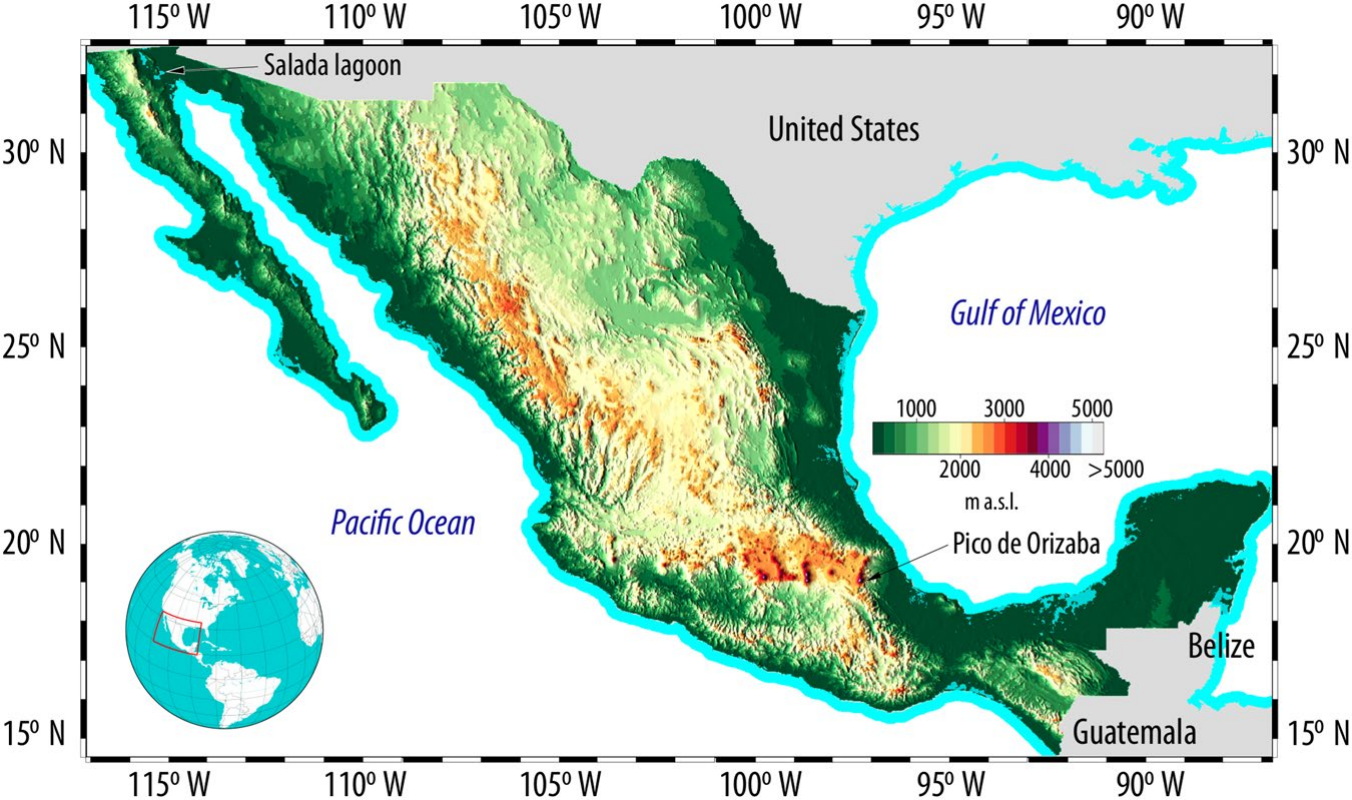
Mexico’s elevation varies from −10 m a.s.l. at the Salada lagoon to 5636 m a.s.l. at the *Pico de Orizaba* volcano. The two mountain ranges (Eastern and Western Sierra Madre) that are parallel to the coast—with elevations above 2500 m a.s.l. are clearly shown. The Digital Elevation Model shown is the *Contínuo de Elevaciones Mexicano* (Mexican Continuous Elevation, or CEM) developed by INEGI and referenced to the U.S. National Geodetic Vertical Datum of 1929 (NGVD29).

Mexico, with an area of 1.9 × 10^6^ km^2^ is surrounded by sea on both its western and eastern sides by the Pacific Ocean and the Gulf of Mexico respectively, and has an elevation that ranges from −10 m a.s.l. on the Salada Lagoon to 5,636 m a.s.l. on *Pico de Orizaba*—its year round snow-capped highest peak (Fig. 1). Two vertical datums have been used in Mexico to obtain orthometric heights: the United States National Geodetic Vertical Datum of 1929 (NGVD29)—and starting in 2015—the North American Vertical Datum of 1988 (NAVD88). Accordingly, the evolution of the vertical geodetic network in Mexico is closely related to the development of the vertical datums on the United States. Mexico developed its National Geodetic Network linked to the United States National Geodetic Vertical Datum of 1929 (NGVD29) through four benchmarks located on the U.S^18^. The United States adopted the NAVD88 as its official vertical datum in the 1990s while Mexico adopted it in 2015^15^. Although Canada participated in the development of the NAVD88, it did not adopt this vertical datum due to concerns related to an east-west sytematic error: the mean sea level of the Pacific Ocean next to Vancouver was around 1.4 m higher than the mean sea level of the Atlantic Ocean next to Halifax^14^. Interested readers can find more information on the development of the aforementioned datums on different publications^2,17,19^.

### Data used

To analyze the difference between the three orthometric heights (*H*_NGVD29_, *H*_NAVD88_, *H*_EGM96_) currently used in Mexico, a total of 141095 PDF files were downloaded from Mexico’s Institute of Geography and Statistics (INEGI) passive geodetic network https://www.inegi.org.mx/app/geo2/rgnp/webpage, which are classified into horizontal or vertical geodetic networks. The horizontal geodetic network is based on static measurements taken with a dual-frequency GPS/GNSS for a minimum duration of three hours—thus providing ellipsoidal heights—and adjusted to Mexico’s Active Geodetic Network (RGNA), which started to operate in 1993^20^. The vertical geodetic network is comprised of monumented benchmarks measured through leveling with a distance of 1 km in both coastal and urban areas, or 2 km in other locations^21^. The basic vertical network—which is used as the basic structure of the national network—was established with a first order class II precision and was developed parallel to the main communication lines of the country^18^. The topographic vertical network—which forms circuits that begin and end on the basic vertical network—was established with a second order class II precision except in mountainous areas, where it was developed with a third order precision, and more details on the development of Mexico’s vertical geodetic network can be found in different publications^18,21,22^.

**Fig. 2:**
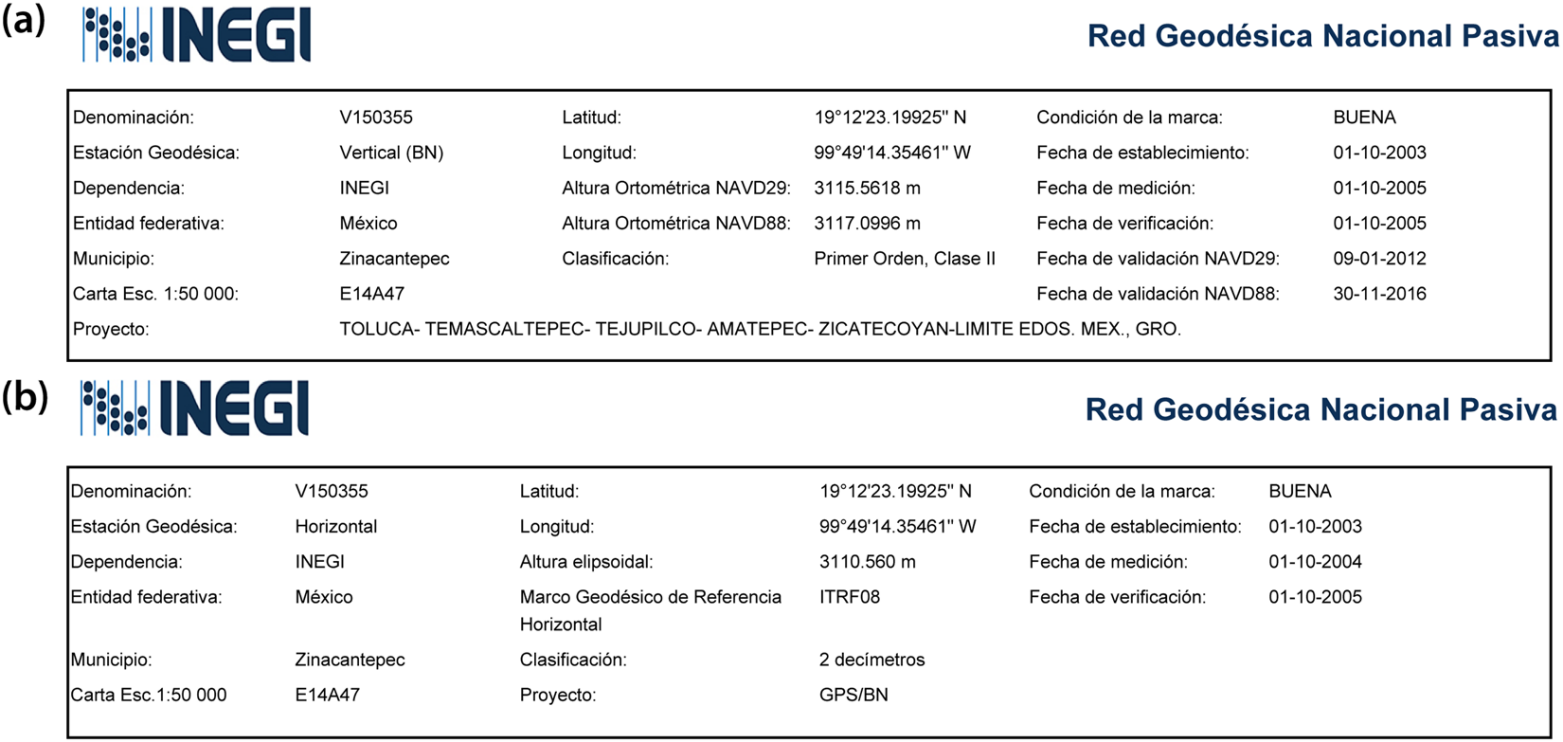
Example of PDF files downloaded from INEGI’s Passive Geodetic Network for (**a**) vertical and (**b**) horizontal geodetic benchmarks. This benchmark was selected because it has orthometric heights for the three vertical datums currently used in Mexico. The difference in orthometric heights for this benchmark is 1.53 m (*H*_NGVD29_ = 3115.56 m, *H*_NAVD88_ = 3117.09 m) while its ellipsoidal height is *h* = 3110.56 m—which has to be converted to a orthometric height. It should be noted that INEGI misnames NGVD29 on the vertical benchmarks—as it uses NAVD29 instead.

From the aforementioned 141095 PDF files, 59395 correspond to the vertical geodetic stations while the remainder 83100 have data for the horizontal geodetic stations—which represent GPS-derived ellipsoidal heights (*h*). It is worth mentioning that although INEGI’s webpage show more geodetic stations, some of them are duplicated. A section of each PDF file is shown in Fig. 2, where Fig. 2(a) shows the orthometric heights referenced to either NGVD29 or NAVD88 along with other information for each vertical benchmark such as its denomination, coordinates and condition, as well as when it was established, measured, verified and validated for each vertical datum. This information is also available for the horizontal benchmarks (Fig. 2(b)), which only have ellipsoidal heights. The remainder section of each file (not shown) provides a sketch in order to find the monumented benchmark. The difference in orthometric heights for the benchmark of Fig. 2 is 1.53 m (*H*_NGVD29_ = 3115.56 m, *H*_NAVD88_ = 3117.09 m) while its ellipsoidal height is *h* = 3110.56 m—as a reminder, this latter height needs to be converted to orthometric height (*H*_EGM96_), which can be done with its respective geoid height (Eq. 1). Although a more recent Earth Geopotential Model (EGM2008) is currently available, this work uses the EGM96 because satellite derived DEMs use EGM96 as their vertical reference surface.

The downloaded PDF files were first processed with the pdf2txt Linux utility in order to create a simple text file for each benchmark. These files where further processed through a series of awk scripts to generate two CSV files—one for each group of benchmarks—that were imported into the GIS GRASS^23^ in order to create a database with a structure previously suggested^24^, which allows the database to be visualized and queried within GRASS, while at the same time further analysis can be undertaken with the statistical software R^25^. The surface transformations were developed using Kriging with External Drift on a local neighborhood (KED_1_) through the use of the R libraries gstat^26^, sp^27^, RPostgreSQL^28^ and rgrass7^29^, while hexscattergram visualization was done with ggplot2^30^ due to the large number of points present.

**Fig. 3:**
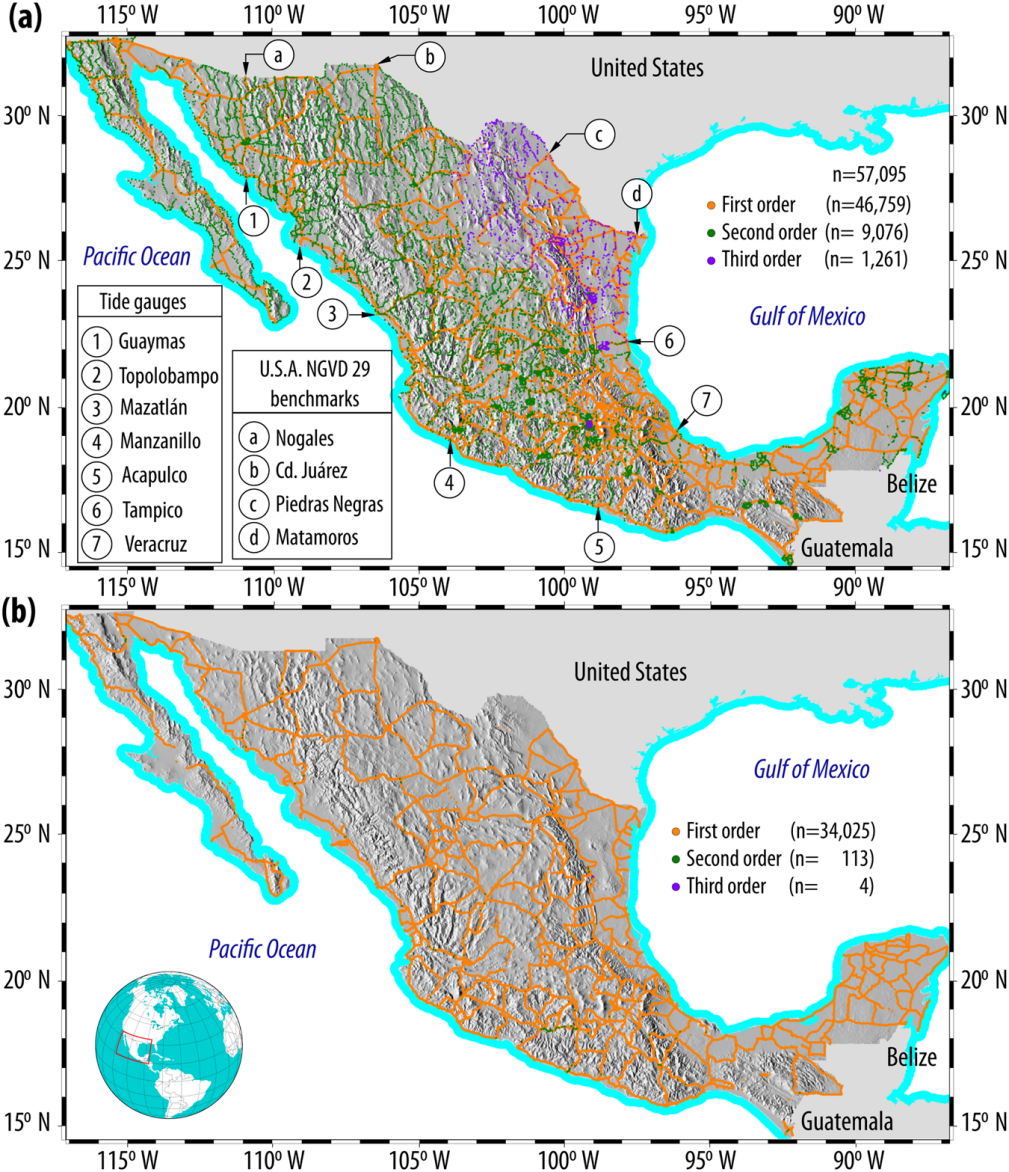
Vertical benchmarks referenced to (**a**) NGVD29 and (**b**) NAVD88. In 1955, Mexico’s geodetic network was linked to the NGVD29 through four U.S. benchmarks: A-680 in Brownsville, TX; T-64 in Eagle Pass, TX, A-110 in El Paso, TX and K-77 in Nogales, AZ, while six mareographic stations were fixed as zero elevation in Mexico at Mazatlán, Manzanillo, Acapulco, Guaymas, Topolobampo and Tampico.

After all the files were processed and imported into GRASS, two vector files were created: one for vertical benchmarks and another one for horizontal stations. The spatial distribution of vertical benchmarks is shown in Fig. 3, where it can be seen that 57095 vertical benchmarks are referenced to the NGVD29 (*H*_NGVD29_, Fig. 3(a)) while only 34142 benchmarks are referenced to the NAVD88 (*H*_NAVD88_, Fig. 3(b)). This Figure also shows the locations where Mexico’s vertical geodetic network was linked to the U.S. NGVD29 through benchmarks A-680 in Brownsville, TX; T-64 in Eagle Pass, TX, A-110 in El Paso, TX and K-77 in Nogales, AZ, along with the six mareographic stations were fixed as zero elevation in Mexico at Mazatlán, Manzanillo, Acapulco, Guaymas, Topolobampo and Tampico^18^.

**Fig. 4:**
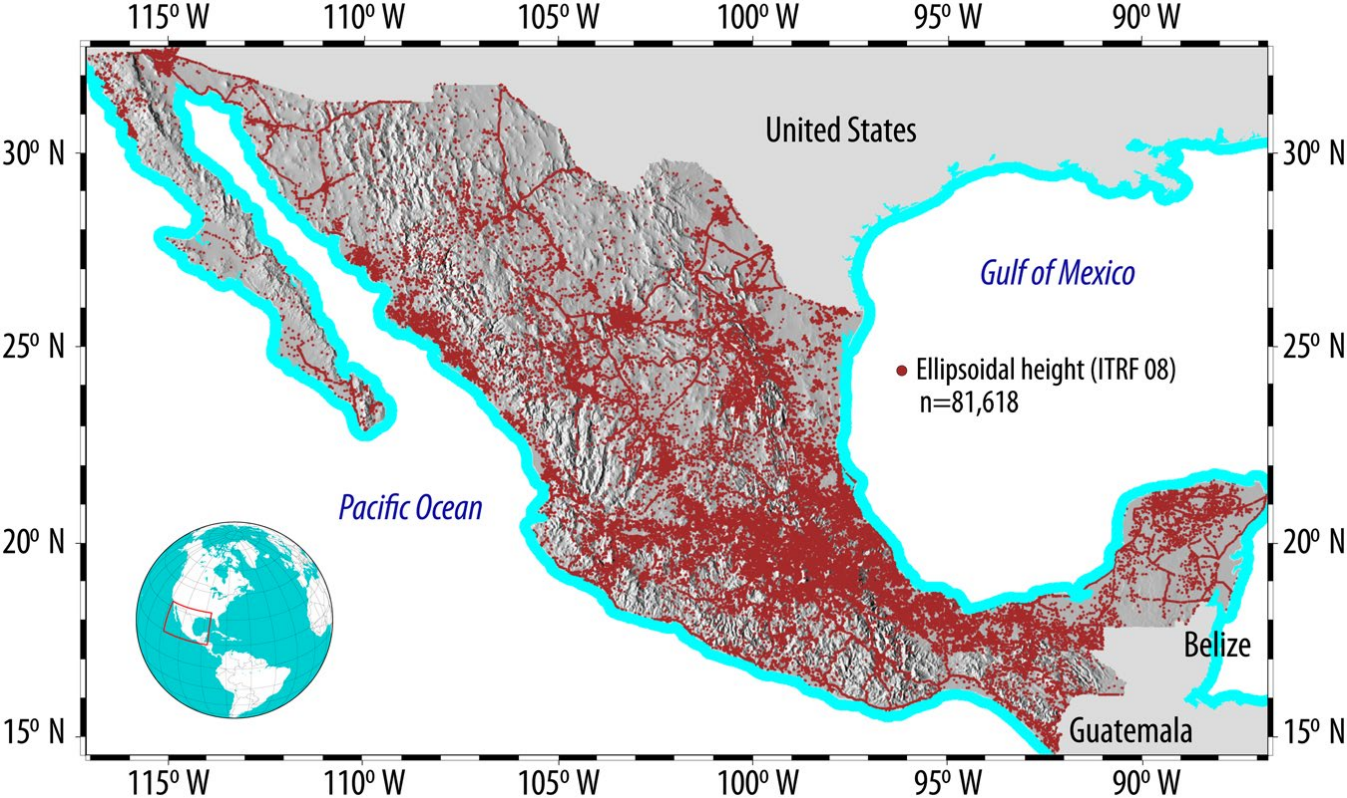
Spatial coverage of Mexico’s horizontal geodetic stations, which consist of GPS/GNSS observations (ellipsoidal heights). These heights are converted to orthometric heights (*H*) through the use of geoid heights.

The spatial distribution of the 83100 horizontal stations—which represent GNSS/GPS-derived ellipsoidal heights (*h*)—is shown in Fig. 4, where a better coverage is appreciated, particularly on Central and East Mexico.

### Surface transformation between *H*_NGVD29_ and *H*_NAVD88_ (Δ*H*_8829_)

**Fig. 5:**
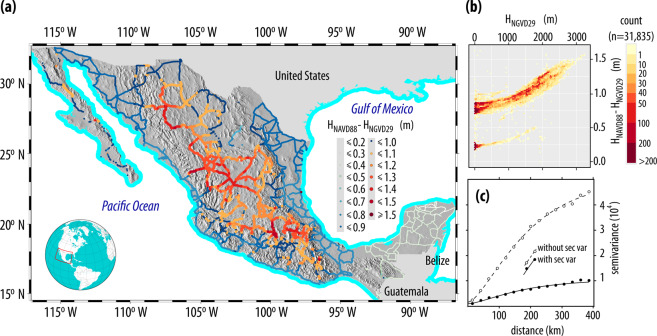
Height difference between NAVD88 and NGVD29 (Δ*H*_8829_): (**a**) spatial distribution in Mexico, (**b**) elevation difference according to orthometric heights referenced to the NGVD29, and (**c**) semivariograms used to represent the spatial correlation of elevation differences with and without using NGVD29 orthometric heights as secondary variable.

As previously mentioned, the goal of this work is to obtain a transformation surface in order to vertically shift NGVD29 to NAVD88 elevations. To achieve this goal, the 31835 vertical benchmarks that have orthometric heights referenced to both the NGVD29 and the NAVD88 were selected, as shown on Fig. 5, where it can be seen that *H*_NAVD88_ − *H*_NGVD29_ for a given vertical benchmark is smaller near the coasts and larger on Mexico’s mountainous regions (Fig. 5(a,b)) and that these differences (Δ*H*_8829_ = *H*_NAVD88_ − *H*_NGVD29_) are positive throughout Mexico—with maximum values of 1.5 m on heights 3000 m above the NGVD29. In comparison, the differences found in the CONUS range from −40 cm to +150 cm^17^, with negative values on the U.S. east coast that increase westwards, reaching their maximum values on the Rockies. It should be mentioned that the differences used in this work are static—as both NGVD29 and NAVD88 are—and that the effects of seismic uplift or subsidence caused by heavy groundwater extraction in central Mexico^31^ are not considered.

To develop the transformation surface (Δ*H*_8829_ = *H*_NAVD88_ − *H*_NGVD29_), Kriging with External Drift on a local neighborhood (KED_l_) was selected as the interpolation algorithm due to the fact that differences in orthometric heights vary according with *H*_NGVD29_ (Fig. 5(b)). Kriging with External Drift (KED) is used when a secondary variable is correlated with the variable of interest^32^—even when these variables exhibit low correlations^33^—and when the value of the secondary variable is known at all data locations and at all locations being estimated^34^. KED was applied on a local neighbourhood (KED_l_) because the mean of the variable to be estimated varies locally and because it is also more computationally efficient^27^. More details on semivariogram modelling and Kriging can be found in well known geostatistics books^34–36^.

The height differences measured at the 31835 vertical benchmarks where both orthometric heights are available (Fig. 5) were first determined and an experimental semivariogram was determined from them. A theoretical semivariogram was adjusted to the experimental semivariogram with and without using *H*_NGVD29_ as secondary variable, and as can be seen on Fig. 5(c) the use of *H*_NGVD29_ as secondary variable reduced the estimated semivariance. A Bessel semivariogram was automatically adjusted using weighted least squares as implemented in gstat^26^ using a cutoff distance of 400 km and Mexico’s Digital Elevation Model (CEM) developed by INEGI—which is referenced to the NGVD29—as secondary variable.

**Fig. 6:**
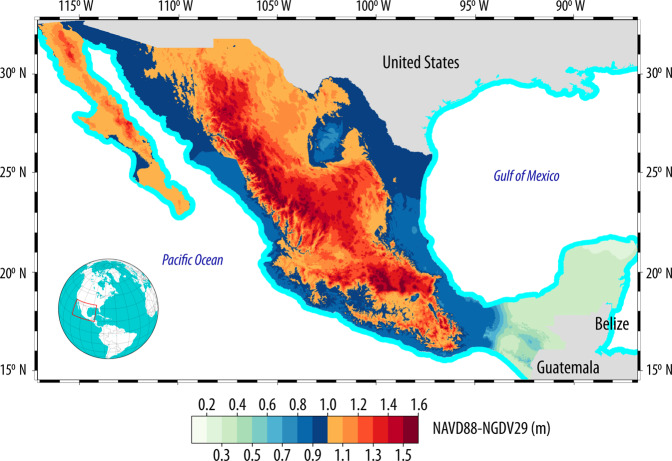
Transformation surface Δ*H*_8829_. In order to obtain a height referenced to the NAVD88, a value obtained from this surface has to be added to the NGVD29 height (*H*_NAVD88_ = *H*_NGVD29_ + Δ*H*_8829_).

The Root Mean Square Error (RMSE), Mean Average Error (MAE) and the Median Absolute Deviation (MAD) of the Δ*H*_8829_ transformation surface were obtained through cross validation—also known as leave-one-out validation—using both gstat and hydroGOF^37^. The aforementioned accuracy measures are reported because they are recommended to assess the accuracy of Digital Elevation Models due to their robustness and distribution free approach to handle outliers^38–40^, and were determined as RMSE = 28.52 mm, MAE = 8.64 mm and MAD = 3.78 mm. The transformation surface Δ*H*_8829_ is shown in Fig. 6, where it can be appreciated that its lowest values appear on the Yucatán Peninsula and along Mexico’s shoreline—except on Baja California, where the difference between orthometric heights are above 1.0 m even on the shoreline.

### Surface transformation between *H*_NGVD29_ and *H*_EGM96_ (Δ*H*_9629_)

**Fig. 7:**
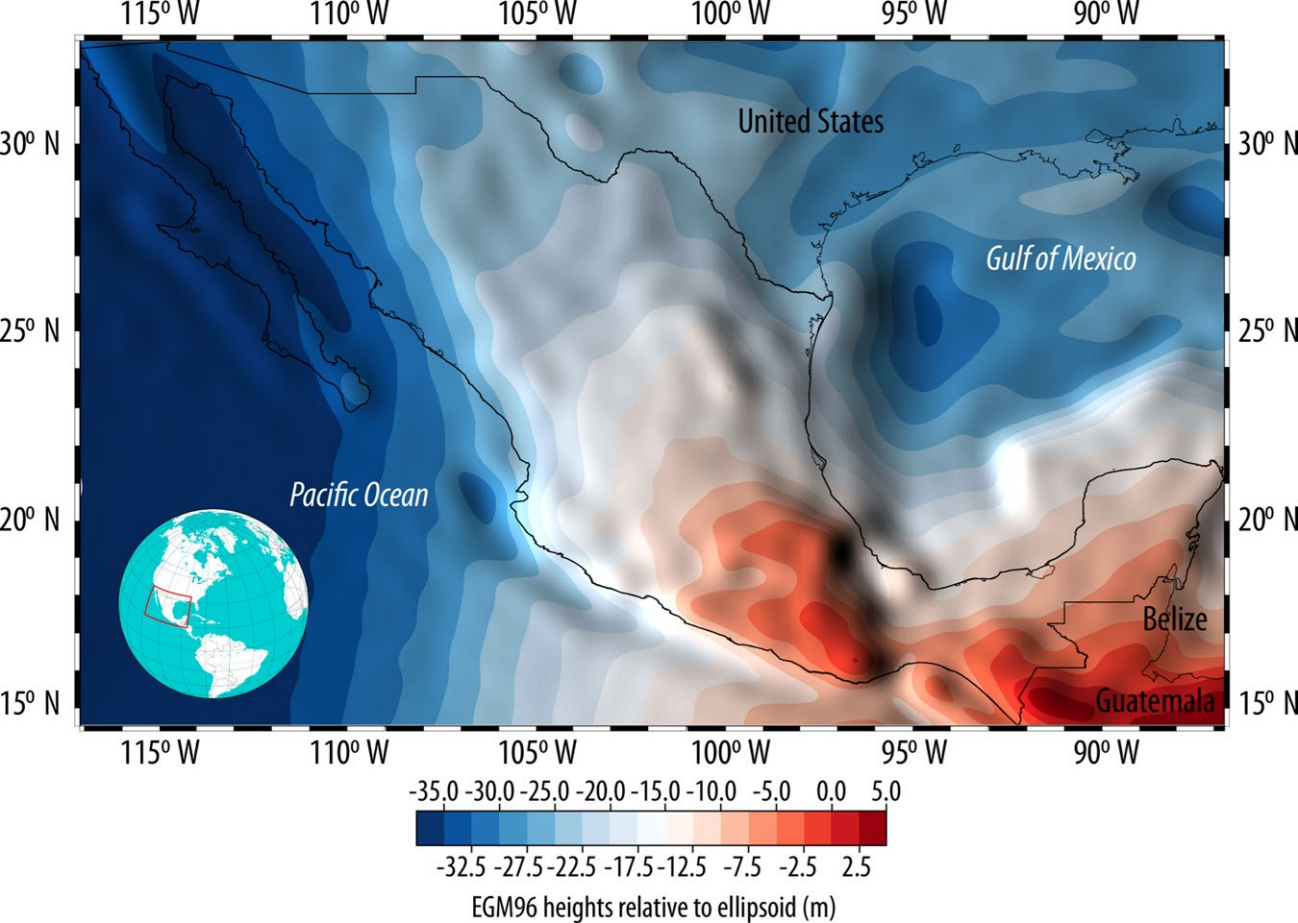
EGM96 heights (*N*_EGM96_) in Mexico at a 1” resolution obtained through bilinear interpolation from the original grid at 15’; this surface represents the vertical datum used in satellite-derived Digital Surface Models (ALOS AW3D30, ASTER and SRTM).

In order to convert the ellipsoidal heights (*h*) measured by INEGI (Fig. 4) to orthometric heights referenced to the Earth Gravitational Model 96 (*H*_EGM96_), the global EGM96 15’ height data (*N*_EGM96_) distributed by the U.S. National Geospatial-Intelligence Agency^3^ was interpolated to a 1” (≈30 m) grid through bilinear interpolation as shown in Fig. 7. This surface was selected as vertical datum because current satellite-derived DEMs (ALOS AW3D30, ASTER and SRTM) are referenced to it and a 1” resolution was assumed adequate to estimate *H*_EGM96_. The geoid heights *N*_EGM96_ at each horizontal benchmark were estimated from the aforementioned surface using GRASS and were added to the ellipsoidal heights (*h*) measured at each horizontal benchmark in order to obtain orthometric heights (*H*_EGM96_) according to Fig. 1. As an example, the ellipsoidal height of benchmark V150355 is *h* = 3110.56 m (Fig. 2(b)), while its EGM96 height is *N*_EGM96_ = −5.705; according to Fig. 1, its orthometric height referenced to the EGM96 is *H*_EGM96_ = 3116.26 m (with *H*_NGVD29_ = 3115.56 m and *H*_NAVD88_ = 3117.09 m).

**Fig. 8:**
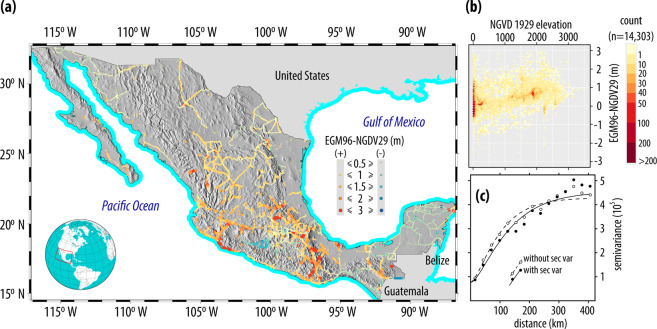
Differences between heights referenced to EGM96 and the NGVD29. A total of 14303 benchmarks have both measurements. (**c**) Semivariograms for the NGVD29-EGM96 elevation differences with and without using *H*_NGVD29_ as secondary variable.

**Fig. 9:**
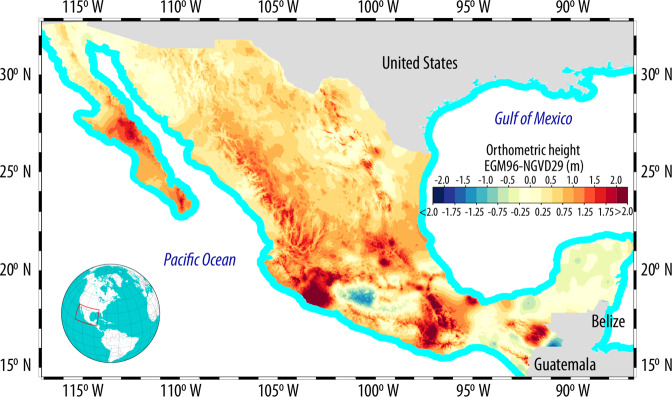
Transformation surface between vertical datums NGVD29 and EGM96. This surface has to be added to a Digital Surface Model referenced to the NGVD29 in order to reference it to the EGM96.

To develop the Δ*H*_9629_ transformation surface, *H*_NGVD29_ were subtracted from *H*_EGM96_ at the 14303 benchmarks that registered both heights, with their spatial coverage shown in Fig. 8(a). These differences show a lower correlation with *H*_NGVD29_ (Fig. 8(a)) than the correlation observed of Δ*H*_8829_ with *H*_NGVD29_ (Fig. 5(a)), which is also evident on the adjusted semivariogram (Fig. 8(c)). Even though Δ*H*_8829_ exhibits a low correlation with *H*_NGVD29_, for this case—just as for the Δ*H*_8829_ surface—the use of Kriging with External Drift on a local neighborhood (KEDl) yielded better accuracy measures than when no secondary variable was considered—a situation that has been reported in the case of daily precipitation^33^. Through cross-validation, the accuracy measures of the Δ*H*_9629_ transformation surface are RMSE = 233.5 mm, MAE = 107.0 mm and MAD = 79.3 mm. The Δ*H*_9629_ transformation surface is shown on Fig. 9, where it can be seen that the datum difference is larger on the central-western region of Mexico, with smaller differences in the Yucatán Peninsula.

To assess the validity of these transformation surfaces, they were added to the original CEM_NGVD29_ in order to obtain CEM_NAVD88_ and CEM_EGM96_ and their accuracy was assessed using the benchmarks that registered orthometric heights referenced to each of the three vertical datums used in Mexico. This analysis is the subject of the following section.

## Data Records

The transformation surfaces developed in this work (Δ*H*_8829_ and Δ*H*_9629_), along with the transformed CEMs (CEM _NAVD88_ and CEM _EGM96_) are available at 10.6084/m9.figshare.11495055 ^41^.

## Technical Validation

The developed transformation surfaces were applied to Mexico’s Continuous Elevation (CEM) developed by INEGI which is referenced to the NGVD29 (*H*_NGVD29_). To shift the CEM’s vertical datum from NGVD29 to NAVD88 the following relationship is applied:

2 $${{\rm{CEM}}}_{{\rm{NAVD88}}}={{\rm{CEM}}}_{{\rm{NGVD29}}}+\Delta {H}_{8829}$$

where Δ*H*_8829_ represents the first transformation surface developed in this work. This datum shift is useful in Mexico in order to use—or compare—the LiDAR DEMs distributed by INEGI which are referenced to the NAVD88 with the CEM. If the CEM needs to be shifted to the EGM96 datum—for example, to compare it with the satellite-derived DEMs—then the following transformation needs to be applied:

3 $${{\rm{CEM}}}_{{\rm{EGM96}}}={{\rm{CEM}}}_{{\rm{NGVD29}}}+\Delta {H}_{9629}$$

where Δ*H*_9629_ represents the second transformation surface developed in this work.

In order to apply both vertical datum shifts, the generated surfaces (Δ*H*_8829_ and Δ*H*_9629_, which were developed at a 1’ resolution (≈1800 m)) were interpolated to a 1” resolution (≈30 m) using bilinear interpolation and added to the CEM_NGVD29_—the two CEMs thus created (CEM_NAVD88_, CEM_EGM96_) are available for download at 10.6084/m9.figshare.11495055. The accuracy of these new CEMs was assessed using the benchmarks that registered orthometric heights referenced to each of the three vertical datums used in Mexico. For these assessments, it was assumed that differences larger than 20 m were outliers, and thus the number of benchmarks used are less than those reported in Figs. 3 and 4. Accordingly, the number of benchmarks used to validate each CEM are ${n}_{{H}_{{\rm{NGVD29}}}}=56961$, ${n}_{{H}_{{\rm{NAVD88}}}}=32950$ and ${n}_{{H}_{{\rm{EGM96}}}}=80584$. Of the aforementioned benchmarks, a total of 31835 have both *H*_NGVD29_ and *H*_NAVD88_, while 14303 have measurements for both *H*_NGVD29_ and *H*_EGM96_.

**Fig. 10:**
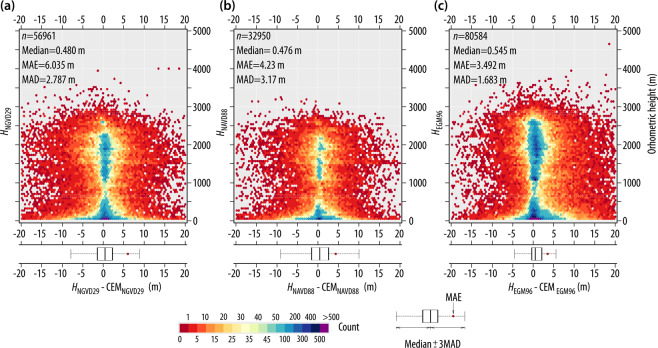
Accuracy assessment of INEGI’s Digital Elevation Model (CEM) using (**a**) Orthometric heights referenced to the NGVD29 and measured at vertical benchmarks (*H*_NGVD29_)—the vertical datum to which the CEM is referenced, (**b**) CEM transformed to the NAVD88 with the use of the Δ*H*_8829_ surface developed in this work and validated using *H*_NAVD88_ measured at vertical benchmarks, and (**c**) CEM referenced to the EGM96 vertical datum and validated using orthogonal heights referenced to EGM96 (*H*_EGM96_). The whiskers extend to three times the Median Absolute Deviation (MAD). MAE represents the Mean Average Error, which is recommended as a more natural measure of average error^38^.

The results of the accuracy assessments for CEM_NGVD29_ (which is the one developed by INEGI), CEM _NAVD88_ and CEM_EGM96_ are shown on Fig. 10(a–c) respectively. This Figure shows that CEM_EGM96_ has the lowest Mean Average Error (MAE_EGM96_ = 3.492 m) and the lowest dispersion—estimated through the Median Absolute Deviation (MAD)—with MAD_EGM96_ = 1.683 m. Of note is the fact that the original CEM (CEM_NGVD29_) has the largest MAE (MAE_NGVD29_ = 6.035 m, MAE_NAVD88_ = 4.23 m). These accuracy measures show that the transformation surfaces Δ*H*_8829_ and Δ*H*_9629_ can be used to undertake a vertical datum transformation due to the fact that the shifted CEMs have a lower Mean Average Error than the original CEM.

## Usage Notes

The transformation surfaces developed in this work, along with the transformed CEMs (CEM_NAVD88_ and CEM_EGM96_) are distributed in GeoTIFF format. Accordingly, these files can be read with any GIS and even in R^25^ using the raster^42^ library.

## Data Availability

The analyses presented in this work were done with the freely available software R^25^ and the transformation surfaces developed are distributed as GeoTIFF files, which can be read in any Geographic Information System software.
